# Dre - Cre Sequential Recombination Provides New Tools for Retinal Ganglion Cell Labeling and Manipulation in Mice

**DOI:** 10.1371/journal.pone.0091435

**Published:** 2014-03-07

**Authors:** Szilard Sajgo, Miruna Georgiana Ghinia, Melody Shi, Pinghu Liu, Lijin Dong, Nadia Parmhans, Octavian Popescu, Tudor Constantin Badea

**Affiliations:** 1 National Eye Institute, NIH, Bethesda, Maryland, United States of America; 2 Biology Department, Babes-Bolyai University, Cluj-Napoca, Cluj, Romania; 3 Institute of Biology, Romanian Academy, Bucharest, Romania; Universidade Federal do ABC, Brazil

## Abstract

**Background:**

Genetic targeting methods have greatly advanced our understanding of many of the 20 Retinal Ganglion Cell (RGC) types conveying visual information from the eyes to the brain. However, the complexity and partial overlap of gene expression patterns in RGCs call for genetic intersectional or sparse labeling strategies. Loci carrying the Cre recombinase in conjunction with conditional knock-out, reporter or other genetic tools can be used for targeted cell type ablation and functional manipulation of specific cell populations. The three members of the Pou4f family of transcription factors, Brn3a, Brn3b and Brn3c, expressed early during RGC development and in combinatorial pattern amongst RGC types are excellent candidates for such gene manipulations.

**Methods and Findings:**

We generated conditional Cre knock-in alleles at the Brn3a and Brn3b loci, *Brn3a^CKOCre^* and *Brn3b^CKOCre^*. When crossed to mice expressing the Dre recombinase, the endogenous Brn3 gene expressed by *Brn3a^CKOCre^* or *Brn3b^CKOCre^* is removed and replaced with a Cre recombinase, generating *Brn3a^Cre^* and *Brn3b^Cre^* knock-in alleles. Surprisingly both *Brn3a^Cre^* and *Brn3b^Cre^* knock-in alleles induce early ubiquitous recombination, consistent with germline expression. However in later stages of development, their expression is limited to the expected endogenous pattern of the *Brn3a* and *Brn3b* genes. We use the *Brn3a^Cre^* and *Brn3b^Cre^* alleles to target a Cre dependent Adeno Associated Virus (AAV) reporter to RGCs and demonstrate its use in morphological characterization, early postnatal gene delivery and tracing the expression of Brn3 genes in RGCs.

**Conclusions:**

Dre recombinase effectively recombines the *Brn3a^CKOCre^* and *Brn3b^CKOCre^* alleles containing its roxP target sites. Sequential Dre to Cre recombination reveals Brn3a and Brn3b expression in early mouse development. The generated *Brn3a^Cre^* and *Brn3b^Cre^* alleles are useful tools that can target exogenously delivered Cre dependent reagents to RGCs in early postnatal development, opening up a large range of potential applications.

## Introduction

Understanding the development and functioning of neuronal circuits is greatly enhanced by mouse genetic tools, which enable the labeling, ablation or functional manipulation of individual neuronal cell types [1]–[3]. Genetically labeled mouse lines help in the definition of neuronal cell types, by allowing an integrated description of morphological, physiological and molecular features of the marked neuronal populations [4]–[7]. In addition, ablation, activation or silencing of specific neuronal populations, most easily achieved by genetic means, greatly helps in understanding their function within the circuit [8]–[11]. Our specific focus, RGCs, the neurons that carry the visual information from the eye to the brain, are a heterogeneous group comprised of about 20 different individual cell types, which have unique combinations of anatomic and physiologic features, and carry out distinct visual functions [12]–[15]. Genetic marking methodologies have greatly advanced our understanding of the functioning of several RGC types. However, a majority of RGC types still cannot be approached by genetic manipulations. To expand the genetic tools enabling us access to RGC populations and RGC types, we are focusing on the three members of the POU4 family of transcription factors, Brn3a, Brn3b and Brn3c, which are expressed in a combinatorial pattern amongst partially overlapping but not identical populations of adult RGC types [16]. Brn3s are expressed early in RGC development, and play diverse roles in RGC type specification [17]–[21]. Thus, Brn3b plays a major role in the specification of a large fraction of RGCs (70%), and seems to be involved in a major fashion in axon formation and a more subtle one in dendritic arbor elaboration. On the other hand, Brn3a although broadly expressed in RGCs, especially in the adult, seems to play a role in specification of a particular subset of RGCs, with small and multistratified dendritic arbors. Brn3c, which is expressed in only three RGC types in the adult, currently has an ill-defined function in RGC development. To understand the exact RGC type distribution of Brn3 transcription factors, we have previously generated conditional reporter knock-in alleles (*Brn3^CKOAP^*), in which the specific endogenous Brn3 gene is selectively ablated and replaced with the histochemical reporter, Alkaline Phosphatase (AP), in a Cre recombinase dependent manner [8]. These alleles enabled us to identify dendritic arbor morphologies and axonal targets within the brain, thus defining RGC types and eventually allowing us to correlate visual functions with particular types of RGCs. They also allowed us to study the fate of RGCs as a group or individually after cell specific ablation of the individual Brn3 gene (*Brn3^AP/−^*), by tracking the axons and dendrites of targeted and hence AP labeled neurons.

Despite these insights, many questions remain unanswered. For instance, although Brn3s are expressed in partially overlapping RGC populations in the adult, it is not clear which fraction of RGC types express each Brn3 gene throughout development, and what role each gene plays in their specification. Also, although the AP reporter is extremely powerful for following morphology during development and through adulthood, as well as diagnosing anatomical defects resulting from genetic manipulations, there are a variety of other questions that will be better addressed by targeting a Cre recombinase to the expression domain of each Brn3 gene. These include lineage tracing, using fluorescent reporters of neuronal activity, performing RGC activation or inhibition using light inducible activation or inhibitory channels, etc. Besides having a very well characterized expression domain and history compared to all other genes in RGCs, Brn3s have the crucial advantage of being the first known RGC markers, expressed as soon as RGCs become postmitotic, at embryonic day E11– E12. Thus, Cre lines generated at the Brn3 loci ought to be extremely useful for the study of RGC development and function.

The Brn3 genes and RGCs however also demonstrate one of the major challenges we have when using genetics to manipulate neuronal cell types. In only very rare instances are gene expression patterns perfectly overlapping with individual RGC types. A majority of RGC associated genes are expressed in several types of RGCs. Thus, in the adult retina, Brn3b and Brn3a seem to be expressed each in 10 distinct RGC types, while Brn3c is expressed only in 3 RGC types. These subpopulations are partially overlapping, with different RGC types expressing either none, one, two or three of the Brn3 genes [16]. It is then a meaningful approach to seek intersectional genetic strategies, based on the Brn3 genes, in order to uniquely label or target a particular RGC type.

The spectrum of gene manipulation tools is continuously expanding, ranging from recombinases to transcriptional regulators [22]–[25]. Strategies based on recombinases are extremely powerful because of their binary switch capability, in which the target gene will be in one state before recombination and a different state afterwards [26]. The most broadly used recombinase is the Cre protein derived from the P1 phage [27]. It is extremely active and specific for its loxP targets sites, and a series of mutant loxP sites are available which react with each other but not with the wild type loxP, resulting in a multitude of gene ablation, reporter induction or gene manipulation strategies [26], [28]–[30].

Two other recombinase – target systems, the FLP recombinase – FRT [31]–[33] and the PhiC31-att [34], [35] system have been developed over the last two decades and are being used in a variety of applications and contexts. They show great specificity for the target sites, but have somewhat reduced efficiencies compared to Cre, although new improved variants have been developed [36]. Several other recombinases from different origins have been recently added to the toolbox [37]–[39]. Amongst these, we chose to use the Dre recombinase, a Cre homolog from the D6 phage [40]. Dre is a site specific recombinase that targets roxP sites of 32 bp containing inverted repeat arms and a central spacer of 4 bases, and has high levels of activity in eukaryotic cells, and transgenic mice [40], [41]. Dre and Cre do not cross react with respect to their roxP and loxP target sites, and based on the high level of homology, their mechanism of action is likely to be very similar [40]. A codon optimized version has been recently developed, and a transgenic mouse line carrying the Dre under the control of the CAG promoter is available from mouse repositories [41]. Thus, it appears that Dre is an excellent candidate for generating combinatorial strategies in conjunction with the more established Cre, Flp and PhiC31 recombinases.

We therefore developed Dre dependent conditional Cre knock-in alleles for the Brn3a and Brn3b loci, trying to achieve several goals. First, we would like to be able to target RGC specific genes as early as feasible during development, taking advantage of the early expression of the Brn3 genes in RGCs. Second, we would like to perform lineage experiments in which the dynamic expression pattern of the Brn3 genes in RGCs is captured. Third, we would like to generate genetic intersections in which only RGCs expressing two of the Brn3 genes are labeled. Fourth, we would like to be able to do this in the context of wild type or mutant Brn3 alleles, and hope to develop Dre expressing drivers which will enable us to do this in a sparse or eye specific manner. Finally, we would like to direct genetic tools for neuronal ablation, activation or inhibition to specific RGC populations.

Unfortunately, due to technical limitations in the targeting constructs, as well as a newly documented expression of Brn3 genes in the germline and/or early embryo, not all these goals will be achievable with the generated alleles. However, besides demonstrating the efficiency and power of the Dre recombinase in mouse genetics, our work brings new insights and useful tools. Brn3a and Brn3b are broadly expressed in early stages of development, but are restricted to correct target populations thereafter, and thus conventional Cre alleles will result in whole embryo recombination when used in conjunction with Cre dependent conditional alleles and reporter lines. However, Cre dependent vectors such as AAV FLEX cassettes can be delivered during relevant stages of RGC development, resulting in accurate and efficient targeting in a Brn3 specific manner.

## Materials and Methods

### Mouse Lines and Targeting

Previously reported mouse lines used in this study are: a) CAG:Dre transgenic line, that drives early ubiquitous expression of the Dre recombinase [41], b) *ROSA26^iAP^*, conditional knock-in line that expresses AP in a Cre recombination dependent manner from the early, ubiquitous ROSA26 locus [42], [43], c) *ROSA26^CreERt^*, expressing a 4-Hydroxytamoxyphen inducible Cre recombinase in an early ubiquitous manner [44], d) *ROSA26^rtTACreERt^*, which expresses Cre recombinase in an early ubiquitous manner under dual pharmacological control of Doxycycline and 4-Hydroxytamoxyphen [42], and e) *Brn3a^CKOAP^* and *Brn3b^CKOAP^*, which allow for the conditional replacement of the *Brn3a* or *Brn3b* loci with AP in a Cre dependent manner [8].

The *Brn3a^CKOCre^* and *Brn3b^CKOCre^* conditional alleles were generated by homologous recombination in mouse embryonic stem cells using analogous strategies to the ones used to generate the *Brn3a^CKOAP^* and *Brn3b^CKOAP^* alleles previously reported [8]. The following changes were made from the original gene structure: a roxP site was inserted in the 5′ UTR 42 bp 50 (for Brn3a) or 98 bp 5′ (for Brn3b) before the initiator codon ATG; 3 repeats of the SV40 early region transcription terminator were added to the 3′ UTR 48 bp (for Brn3a) or 340 bp (for Brn3b) 3′ of the Brn3 translation termination codon, followed by a second roxP site and the coding region of the Cre recombinase. A positive selection cassette (PGK-Neo), flanked by FRT sites, followed the Cre cDNA and was subsequently removed by crossing to mice expressing FLP recombinase in the germline. All mice used were of mixed C57Bl6/SV129 background. All mouse handling procedures used in this study were approved by the National Eye Institute Animal Care and Use Committee (ACUC) under protocols NEI 640, NEI 651 and NEI 652.

### Doxycycline, 4-hydroxytamoxifen (4HT) and AAV Treatments

Induction of recombination in the *ROSA26^rtTACreERt^*; *Brn3b^CKOAP^* mice was achieved by administering doxycycline food (200 mg/gr chow) to pregnant females on alternating days between embryonic days E2– E10 and one 12.5 µg 4HT IP injection at E10. For viral intraocular injection, postnatal day 0 (P0) pups were anesthetized by hypothermia for 30 seconds, a slit was cut in the eye lid, and roughly 0.2 µl of a mix of two viral vectors including AAV1.CAG.FLEX.tdTomato.WPRE.bGH (at 5.7 * e8, Gene Therapy Program at the University of Pennsylvania, Philadelphia, PA) expressing red fluorescent tdTomato in a Cre recombination dependent manner, and a AAV1.CAG.GFP, expressing GFP in a Cre independent manner (1*e8 GFP virus, gift of Dr. Peter Colosi and Zujhian Wu), were injected, using a femtojet (Eppendorf) fitted with a pulled glass capillary. Pups were then returned to their mothers and collected at P3, P7 and adult.

### Histology and Immunohistochemistry

Alkaline Phosphatase histochemistry of retina flat mounts and brain vibratome sections were performed as previously described [8], [16], [44], [45]. Wholemount embryos of genotypes indicated in the text were collected from timed pregnancies at E9.5 and E12.5, immersion fixed overnight in 4% Paraformaldehyde at 4 C, heat inactivated for one hour at 65 C in PBS, and then AP histochemistry was developed for 2–4 hours. Eyes from virus-infected pups or adult mice were enucleated, prefixed in 2% PFA for 15 minutes at room temperature, the cornea was cut out, the lens removed, and the eye cups were further fixed for 30 minutes. The retinas were then separated from the rest of the eye tissues, and flat mounted in Aquamount. For immunofluorescence experiments, eyes were fixed for 30 minutes in 2% PFA, equilibrated in sucrose and embedded in OCT. Endogenous fluorescence was detected in the red channel for tdTomato, while Brn3a, Brn3b (own production, previously reported [46] and NFL (Chemicon – Millipore, AB9568) were immunodetected with rabbit antibodies and Alexa 647 Donkey anti-rabbit secondary (Molecular Probes – life technologies). The Alexa 647 signals, detected with an infrared filter were pseudocolored in green for double immunofluorescence experiments. Imaging for all retina preparations were performed with a Zeiss AxioImager.2, with a 5x objective for survey of the entire retina, or 20x, 40x or 63x magnification and the Apotome for immunofluorescence detection, and collected with either color or black-and-white Zeiss Axiocams. Brain sections were imaged with a color Zeiss Axiocam adapted onto a Zeiss Discovery. V8 stereomicroscope. Images were imported with custom written ImageJ plugins, LUTs corrected and, where necessary z-stack projections were generated under ImageJ.

### Statistical Analysis

For each immunostaining – genotype combination, 3 to 5 pictures at 20x magnification were taken from retina sections of multiple animals. For each image, the numbers of marker, tdTomato or double positive cells were expressed as percentages of DAPI positive cells in the section, and represented as Box-Whisker plots. Explanation of Box Whisker plots: the tops and bottoms of each “box” are the 25th and 75th percentiles of the samples, respectively. The distances between the tops and bottoms are the interquartile ranges. The line in the middle of each box is the sample median. Whiskers are drawn from the ends of the interquartile ranges to the furthest observations within the whisker length (the adjacent values). Student T tests under assumption of normal distribution and Kolmogorov-Smirnov (KS2) tests for comparison of two data sets of unknown distributions were performed, with comparable results for the relevant comparisons (tdT only versus tdT & Marker), using Matlab (The Mathworks, Inc.). All n, averages and standard deviations are reported in Tables (S1 and S2).

## Results

### Generation of *Brn3a^CKOCre^* and *Brn3b^CKOCre^* Conditional Cre Knock-in Alleles

**Figure 1:**
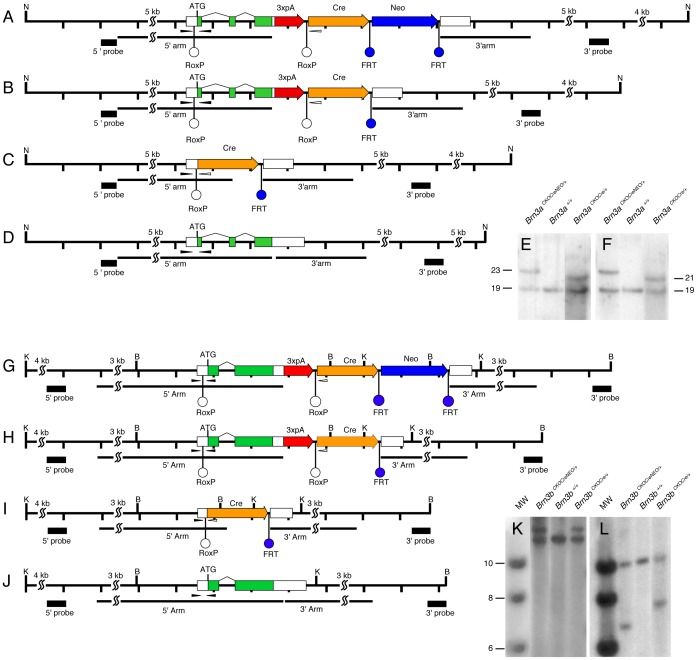
Conditional Cre knock-in constructs at the Brn3a and Brn3b loci. Diagrams represent the targeting strategy and recombination steps for the Brn3a (A–D) and Brn3b (G–J) locus. D, J, wild type configuration, A,G, locus after gene targeting, B,H, locus after removal of the Neo selection cassette by cross with germline Flp recombinase expressing mice, C,I, locus after removal of the endogenous gene by Dre mediated recombination, resulting in expression of the Cre recombinase gene from the original transcription start site of the Brn3 gene. Tick marks represent 1 kb distances, except for contracted longer stretches marked with SS marks. Green and white boxes represent coding regions and UTRs respectively, and splice junctions are indicated by angled black lines. Homologous recombination targeting arms are indicated by black lines, and southern blot probes as black boxes under the locus. Diagnostic restriction enzymes are represented as capital letters: Nde I (N), Kpn I (K), and Bam HI (B). A triple repeat of the SV40 polyA is inserted in the 3′UTR of the targeted locus, and indicated by a red thick arrow. RoxP sites (white circles), flank the endogenous gene while FRT sites (blue circles), flank the PGK-Neo Cassette. Genotyping primers (thin black arrowheads) are placed in the 5′ UTR of the loci, and encompass the ATG in the wild type and ATG and 5′ roxP site in the targeted locus. After Dre recombination (C, I) the reverse strand black primer is lost, and correct localization of the Cre can be tested by the black and white pair of primers indicated (see also figure 2). Southern blot fragment lengths are as follows: for Brn3a, the Nde I fragment is recognized by both 5′ and 3′ probes, and is 19116 bp for the wild type, 23542 for the targeted locus, and 21637 bp for the targeted locus after Flp mediated Neo cassette removal; for Brn3b, Kpn I digests are tested with the 5′ probe, recognizing fragments of 12624 bp in wild type, and 14388 bp in the targeted construct, before and after Neo cassette removal; Bam HI digests, tested with the 3′ probe yielding characteristic fragments of 10140 bp in the wild type, 6772 bp in the targeted locus, and 7682 bp after Neo removal. E, F, Genotyping by southern blot for the 5′ (E) and 3′ (F) ends of the Brn3a targeting event. For each E and F, targeted locus (A), wild type control (D), and targeted locus after Neo removal (B) are shown. K, L Genotyping by southern blot for the 5′ (K) and 3′ (L) ends of the Brn3b targeting event. For each K and L, molecular weight (MW) markers are followed by targeted locus (G), wild type control (J), and targeted locus after Neo removal (H).

Based on the previously described *Brn3^CKOAP^* alleles, we have generated two new targeting constructs in which the loxP sites were replaced with roxP sites and the AP open reading frame with a Cre recombinase (Fig. 1). Thus, the coding region of each Brn3 gene is comprised between roxP sites, and followed by the cDNA for the Cre recombinase. The loci are designed such that, after Dre mediated recombination between the two roxP sites, the Brn3a or Brn3b gene is removed and replaced with the Cre recombinase, which will now be expressed under the control of the respective endogenous Brn3 locus. It carries most of the 3′ UTR of the respective Brn3 mRNA, and is expected to faithfully reproduce the expression profile of the endogenous Brn3 gene. The targeting constructs also contain a PGK-Neo selection cassette flanked by FRT sites.

For each Brn3a and Brn3b the diagrams in Figure 1 show the wild type loci (Fig. 1 D, J) and the targeted alleles before (Fig. 1 A, G) and after (Fig. 1 B, H) removal of the FRT- PGK-Neo-FRT selection cassette via Flp mediated recombination. Figure 1, C and I show schematics of the Brn3a and Brn3b loci after Dre mediated recombination, resulting in loss of the Brn3 coding exons, and their replacement with the Cre recombinase. Correct targeting and removal of the PGK-Neo selection cassette was tested for both Brn3a (Fig. 1 E and F) and Brn3b (Fig. 1 K and L) by southern blotting of genomic DNA with 5′ and 3′ probes, and the expected fragments confirmed. Both *Brn3a^CKOCre^* and *Brn3b^CKOCre^* lines can be maintained as homozygotes and are viable and fertile, suggesting, at least for the Brn3a locus, that the endogenous Brn3a gene expression has not been majorly disrupted by the conditional targeting event.

### Testing Sequential Dre to Cre Recombination in CAG:Dre; *Brn3^CKOCre^*; *ROSA26^iAP^* Triple Transgenic Mice

**Figure 2:**
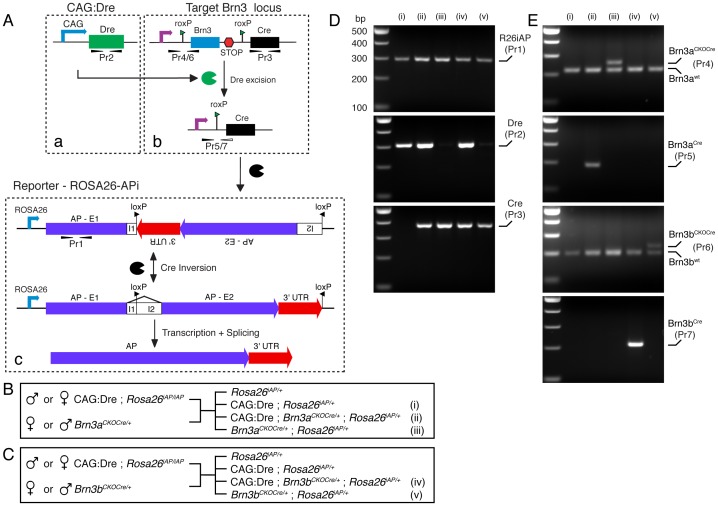
Recombination strategy used to test sequential Dre and Cre recombination. A, Genetic loci and recombination events. The CAG:Dre transgene (a), expresses ubiquitously Dre recombinase that targets the roxP sites of the *Brn3^CKOCre^* locus (b), removing the endogenous exons of the Brn3 gene and replacing them with the Cre open reading frame. After Dre recombination, primer pairs Pr4 (Brn3a) or Pr6 (Brn3b) are rendered nonproductive by the loss of the reverse strand primer and novel primer pairs Pr5 (Brn3a) and Pr7 (Brn3b) are generated, between the forward strand primer remaining 5′ of the roxP site in the 5′UTR and a reverse strand primer placed in the Cre gene. The Brn3 locus starts expressing the Cre gene, which targets the inverted loxP sites of the *ROSA26^iAP^* locus (c), reversing the inverted exon, resulting in productive transcription of the AP protein. B, C, Crosses generating the triple transgenic mice and the appropriate controls, for *Brn3a^CKOCre^* and *Brn3b^CKOCre^* alleles. Note that, to control for the germline expression of the *Brn3^CKOCre^* loci, the crosses were performed in both male to female combinations. D, PCR genotyping demonstrating the presence of the AP (primer pair Pr1), Dre (primer pair Pr2) and Cre (primer pair Pr3) genes, in the various genetic knock-in combinations. E, PCR reactions demonstrating the insertion of the roxP site in the 5′ UTR of the *Brn3a^CKOCre^* (iii, Pr4) and *Brn3b^CKOCre^* (v, Pr6) loci. Note that all samples have one wild type chromosome, showing a band of 220 bp for Brn3a (Pr4) and 200 bp for Brn3b (Pr6), but only the Dre – negative *Brn3a^CKOCre^* (iii) or *Brn3b^CKOCre^* (v) genetic combinations show a roxP insertion shifted by ∼ 30 bp up (top band). In contrast, in the triple transgenic combinations (ii and iv) in which Dre recombination has occurred, the roxP insertion band is removed (top band, Pr4 and Pr6), and the Brn3-Cre reactions (Pr5 and Pr7) become positive.

To date, only one publication reports successful Dre recombination in transgenic mice [41]. To test whether our conditional alleles are indeed susceptible to Dre mediated recombination, we crossed them with the ubiquitous Dre driver line, CAG:Dre, described by Annastasiadis and coworkers [41], and our previously published Cre reporter line, *ROSA26^iAP^*, generating triple transgenic animals as well as appropriate double transgenic controls (Fig. 2). The expected succession of recombination events (Fig. 2A) involves early/germline expression of the Dre recombinase followed by conditional ablation of the Brn3 gene and induction of Cre expression from the Brn3 locus, which will be reported by expression of the Cre dependent AP, transcribed from the ubiquitous ROSA26 locus. Detection of the recombination event was done by two diagnostic genotyping primer pairs (Fig. 1, C, D, I, J and Fig. 2 A, D, and E). The first set of primers, shown as black arrow heads in Fig. 1, D and J and numbered as primer pairs Pr4 for Brn3a and Pr6 for Brn3b in Figure 2Ab and E, consists of a forward primer placed in the 5′UTR of the respective Brn3 gene, upstream of the insertion site of the roxP sequence, and a reverse primer, inserted in the open reading frame of the first exon, so downstream of the insertion site of the roxP. These primers will detect the wild type configuration of the loci, and a relative upward shift of 32 bp for the roxP insertion in *Brn3a^CKOCre/+^; ROSA26^iAP/+^* and *Brn3b^CKOCre/+^; ROSA26^iAP/+^* mice (Fig. 2E, iii and v). However, in the triple transgenic CAG:Dre; *Brn3a^CKOCre/+^; ROSA26^iAP/+^* and CAG:Dre; *Brn3b^CKOCre/+^; ROSA26^iAP/+^* mice, Dre recombination results in removal of the sequence between the two roxP sites, and hence of the reverse primer of primer pairs Pr4 and Pr6, and thus only the shorter product, generated from the wild type Brn3 allele, can be detected (Fig. 2E, ii and iv). In contrast, bringing the Cre recombinase cDNA in close apposition to the Brn3 5′UTR generates a novel PCR product between the forward primer and a reverse primer, placed in the Cre recombinase (black and white arrowheads in Figure 1, C and I and primer pairs Pr5 and Pr7 in Figure 2Ab and E, ii and iv). The specific removal of the diagnostic roxP products (Pr4 and Pr6) and generation of the Brn3-Cre products (Pr5 and Pr7), in CAG:Dre; *Brn3^CKOCre/+^; ROSA26^iAP/+^* mice but not in *Brn3^CKOCre/+^; ROSA26^iAP/+^* controls, (Fig. 2E) demonstrates that Dre recombination was successful and complete in these animals.

### Sequential Dre to Cre Recombination Reveals Early/ubiquitous Expression of Cre from the Endogenous Brn3a and Brn3b Loci

**Figure 3:**
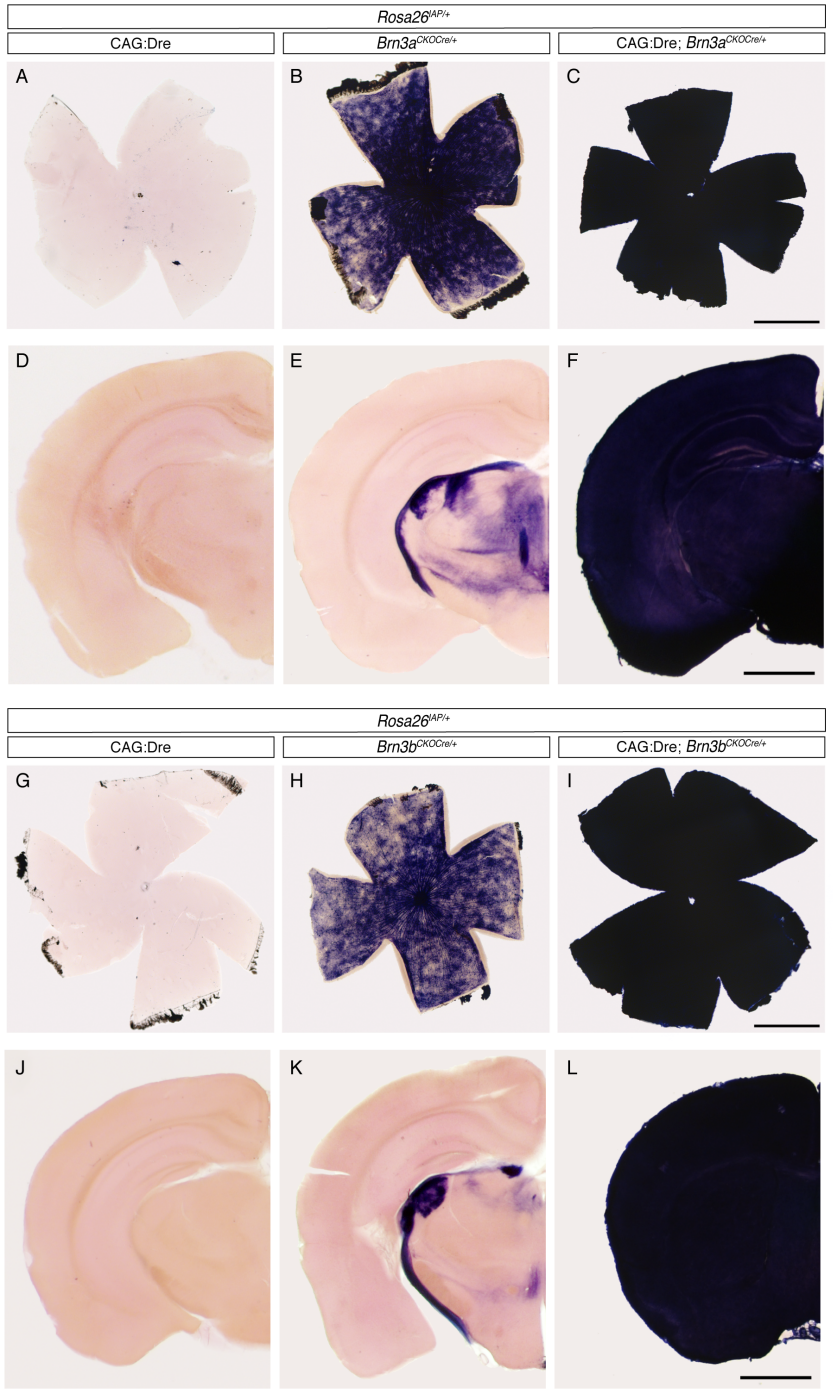
Sequential Dre to Cre recombination suggests ubiquitous Cre expression from the Brn3a and Brn3b loci. A–C, G–I, Adult retina flat mounts and D–F, J–L, hemispheres from coronal brain sections of mice with indicated genotypes. Note that, in CAG:Dre; *ROSA26^iAP^* mice, germline Dre expression does not result in induction of AP positivity from the Cre dependent *ROSA26^iAP^* locus (A, D - n = 4; G, J – n = 6). However, *Brn3^CKOCre^; ROSA26^iAP^* tissues show a reduced level of mosaic recombination in RGCs, and corresponding projection areas in the brain (B, E - n = 9; H, K – n = 13). In contrast, tissues from CAG:Dre; *Brn3^CKOCre^; ROSA26^iAP^* mice (C, F - n = 4; I, L – n = 13) show complete conversion to AP positivity, suggesting that the sequential Dre to Cre to AP recombination happened in the totality (or a vast majority) of the tissue. Scale bars in C, F, I and L are 1 mm.

**Figure 4:**
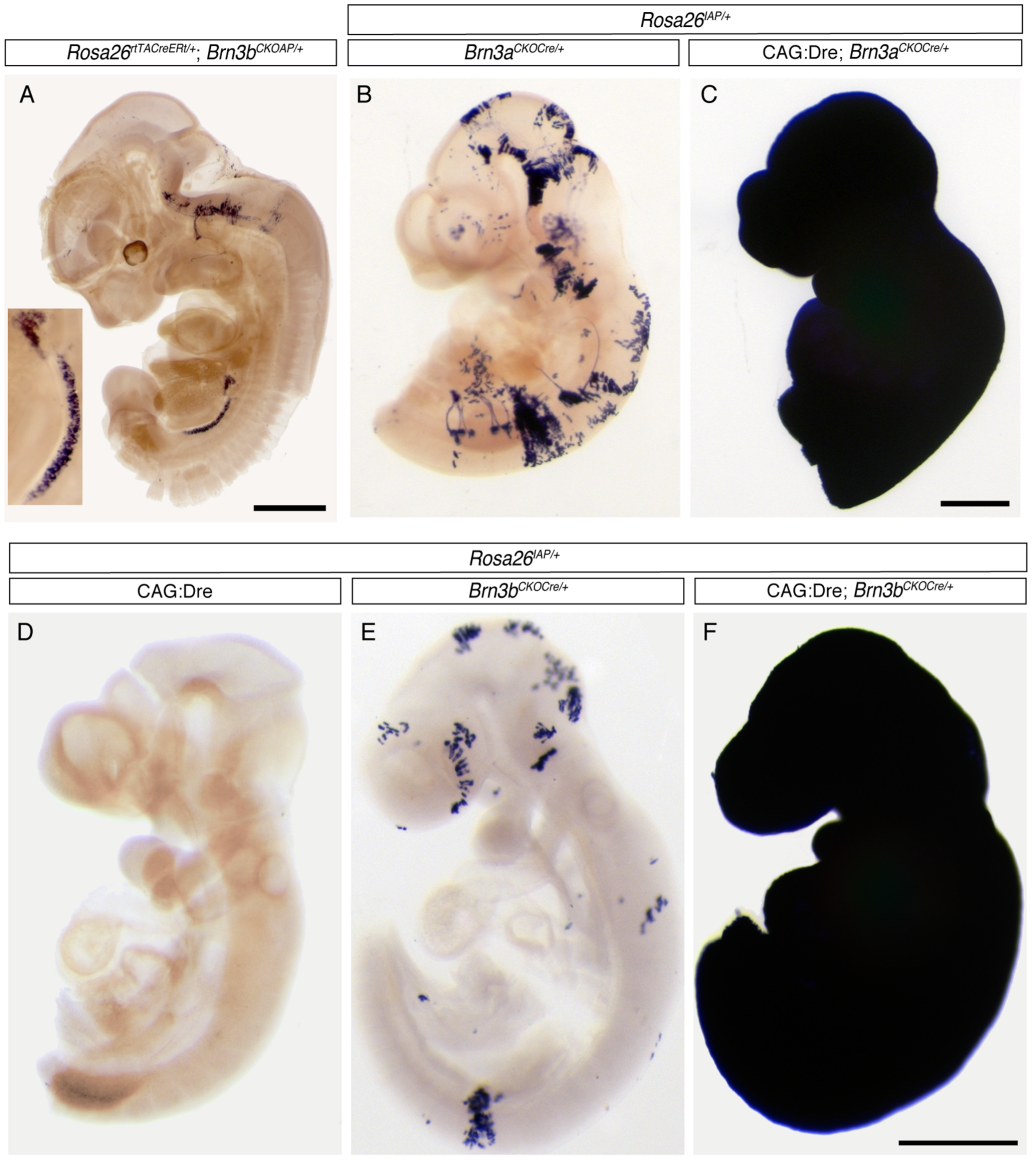
Whole tissue sequential Dre to Cre recombination in CAG:Dre; *Brn3^CKOCre^; ROSA26^iAP^* mice happens before E9.5. A, wholemount staining of E12.5 *ROSA26^rtTACreERt^*; *Brn3b^CKOAP^* embryo demonstrating *Brn3b^AP^* labeling of the germinal ridge. Inset shows 8x magnification of the gonad. B, C, wholemount E9.5 embryos showing complete AP recombination in CAG:Dre; *Brn3a^CKOCre^; ROSA26^iAP^* mice (C, n = 6) and sparse mosaic recombination in *Brn3a^CKOCre^; ROSA26^iAP^* (B, n = 9) controls. Whereas no AP positive cells are visible in E9 CAG:Dre; *ROSA26^iAP^* embryos (D, n = 4), sparse recombination can be seen in *Brn3b^CKOCre^; ROSA26^iAP^* (E, n = 6) and full recombination in CAG:Dre; *Brn3b^CKOCre^; ROSA26^iAP^* (F, n = 7) littermates. Scale bars in A, C, F are 0.5 mm.

We tested the expression pattern of the AP reporter in our triple transgenic CAG:Dre; *Brn3a^CKOCre/+^; ROSA26^iAP/+^* and CAG:Dre; *Brn3b^CKOCre/+^; ROSA26^iAP/+^* mice by histochemical staining of flat mount retinas and coronal brain sections (Figure 3). To our surprise, both retinas and brains of adult triple transgenic animals were completely and homogeneously stained, suggesting ubiquitous recombination of the *ROSA26^iAP/+^* Cre reporter locus (Fig. 3C, F, I, L). In contrast, tissues from CAG:Dre; *ROSA26^iAP/+^* littermates, stained and processed under identical condition, showed no staining, confirming previous results that the Dre recombinase by itself cannot mediate recombination of loxP sites. However, the retinas of *Brn3a^CKOCre/+^; ROSA26^iAP/+^* and *Brn3b^CKOCre/+^; ROSA26^iAP/+^* littermates showed a number of RGCs labeled with AP (Fig. 3 B, H), suggesting Dre independent production of Cre from the *Brn3a^CKOCre/+^* and *Brn3b^CKOCre/+^* loci. Consistent with this, brain sections from both *Brn3^CKOCre/+^; ROSA26^iAP/+^* mice show labeling of the RGC and other Brn3-expressing axonal tracts and nuclei (Fig. 3 E, K). Although the AP staining observed in the double transgenic *Brn3^CKOCre/+^; ROSA26^iAP/+^* retinas and brains marks only a few percent of previously described Brn3 target neurons, these numbers make the two alleles less useful for sparse recombination approaches. We were also intrigued by the apparently complete neuronal recombination pattern seen in the triple transgenic animal. This is unlikely explained by a defect in our targeting design or execution, as the previously reported *Brn3a^CKOAP/+^* and *Brn3b^CKOAP/+^* alleles, identical from a gene regulation perspective, have been shown to correctly reflect the endogenous expression pattern of the *Brn3a* and *Brn3b* genes in several neuronal targets [8], [16], [45]. In addition, both Southern blot and PCR evidence suggest that the homologous recombination and Neo selection removal happened correctly (Fig. 1 and 2), and similar ubiquitous recombination patterns were observed with two independent *Brn3^CKOCre/+^* lines. Moreover, we bred out the recombined *Brn3b^Cre/+^* allele (Fig. 1H) from CAG:Dre; *Brn3b^CKOCre/+^* offspring, and crossed it to *ROSA26^iAP/iAP^* animals to generate *Brn3b^Cre/+^; ROSA26^iAP/+^* mice, which had full recombination patterns identical to the triple transgenics (data not shown), suggesting that this effect is not dependent on the Dre to Cre interaction. A previous report had suggested expression of Brn3a and Brn3b in both the male and female germline (ref [47], see Fig. 1 for evidence of Brn3a and Brn3b expression in both testis and ovary). We therefore performed the crosses resulting in generation of triple transgenic animals, CAG:Dre; *Brn3^CKOCre/+^; ROSA26^iAP/+^*, providing the *Brn3^CKOCre^* alleles from either the male or female parent, with similar results being obtained (Fig. 2 B and C). To test how early the Dre to Cre sequential recombination occurred, we stained E9–E10 CAG:Dre; *Brn3^CKOCre/+^; ROSA26^iAP/+^*, triple transgenic embryos in whole mount and found that indeed the entire embryo was AP positive (Fig. 4C, F), whereas the CAG:Dre; *ROSA26^iAP/+^* and *Brn3^CKOCre/+^; ROSA26^iAP/+^* control embryos showed no or sparse recombination respectively (Fig. 4B, D, E). Finally, we stained E12.5 *ROSA26^CreERt/+^; Brn3a^CKOAP/+^* (not shown) and *ROSA26^rtTACreERt/+^; Brn3b^CKOAP/+^* (Fig. 4A) embryos, in which sparse recombination had been induced as previously [16], [42], and noticed abundant AP expression in the germ ridge (see ref [48] for a comparison). Thus we can conclude that *Brn3a^CKOCre/+^* and *Brn3b^CKOCre/+^* alleles are expressed in ubiquitous fashion throughout the embryo at a time point before E9, and most likely in the germline of both males and females.

### *Brn3a^Cre^* and *Brn3b^Cre^* are Driving RGC Specific Recombination at Postnatal Ages

**Figure 5:**
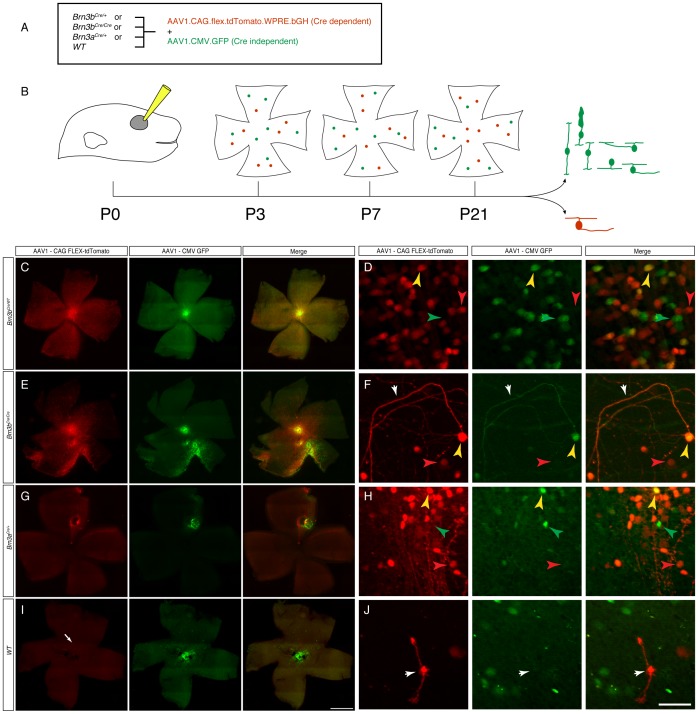
Retinal expression of viral Cre dependent reporters after postnatal day 0.5 (P0) intraocular infection. A, Genetic backgrounds and viruses used. *Brn3b^Cre/+^*, *Brn3b^Cre/Cre^*, *Brn3a^Cre/+^*, or *Brn3b^+/+^* littermate pups were infected with a combination of Cre dependent AAV1.CAG.flex.tdTomato.WPRE.bGH virus and a constitutively expressed, Cre independent AAV1.CAG.GFP virus. B, Experimental time lines. Eyes of P0 pups were injected, and retinas were analyzed at either P3, P7 or after P21. Cells infected with the Cre independent virus should appear green, while Cre positive cells infected with the Cre dependent virus should appear red. Results from this experiment are shown in figures 5, 6 and 7. C, E, G, and I, flat mount preparations of adult retinas of indicated genotypes, demonstrating extensive expression of (Cre dependent) tdTomato red staining in *Brn3b^Cre/+^* (n = 6), *Brn3b^Cre/Cre^* (n = 4) or *Brn3a^Cre/+^* (n = 12) retinas, and only very isolated expression in Cre negative, *WT* retinas (n = 3 for the Brnb litters and n = 4 for the Brn3a litters). Note that in some cases, red and green fluorescence are evenly distributed over the entire retina, while in others there is an apparent segregation of red and green fluorescence, most likely by subretinal distribution of the Cre independent, AAV1.CAG.GFP virus (see figure 6). White arrow in I points at one of the few red cells labeled in the *WT* retinas, shown enlarged in the inset in J. D, F, H, and J represent higher magnifications of retinas shown in C, E, G, and J respectively. Left panels are tdTomato, red fluorescence, middle panels are GFP, green fluorescence, and right panels are merge channels. In all panels, red arrowheads point at tdTomato positive, green arrowheads at GFP positive, and yellow arrowheads at double positive cells. White arrowhead in F labels wandering axons characteristic of Brn3b null (*Brn3b^Cre/Cre^*) retinas. White arrowhead in J points at the tdTomato positive cell in I, most likely a Müller Glia. Scale bars in I = 1 mm and J = 50 µm.

**Figure 6:**
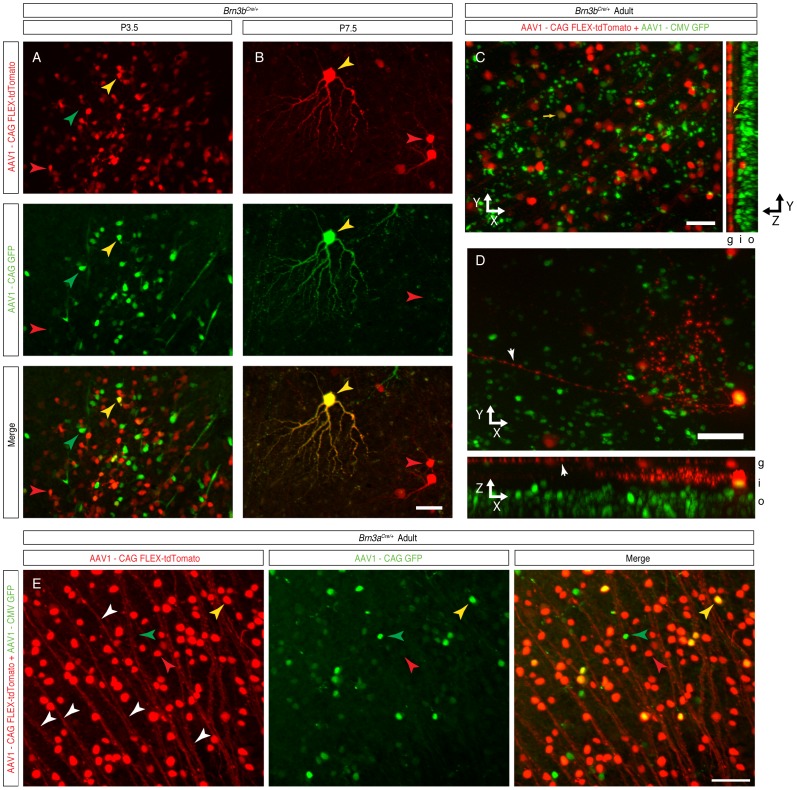
AAV1-FLEX-tdTomato infection at P0 results in RGC specific tdTomato expression, which is already detectable at P3.5 and P7.5. Retina samples are from P3.5 (A), P7.5 (B) pups or adults (C–E). Panels in A, B are tdTomato (top), GFP (middle), and merged (bottom) images. Panels A-D are from *Brn3b^Cre/+^*, and E from from *Brn3a^Cre/+^* mice, infected at P0 after the protocol described in figure 5 A, B. Red, green and yellow arrowheads point at examples of red and green single positive or double positive cells. Note that the dendritic arbor of the double positive cell in B is clearly visible in both red and green channels. C, Projections along the z direction (left) and x direction (right) of a stack from a densely labeled *Brn3b^Cre/+^* retina, showing an overwhelming majority of red fluorescent cell bodies stratified in the GCL (g) layer, whereas abundant numbers of green fluorescent bodies are seen throughout the Inner and Outer Nuclear Layer (i and o). D, Projections along the z direction (top) and y direction (bottom) of a stack from a sparsely infected *Brn3b^Cre/+^* retina showing a displaced RGC, with its axon (white arrowheads) and dendritic arbor, seen both from the flat mount and transversal perspective. E, z projection of a stack from a densely labeled *Brn3a^Cre/+^* retina, showing single and double labeled cell bodies, and tdTomato labeled RGC axons (white arrowheads). Scale bars in B, C, D and E = 50 µm.

**Figure 7:**
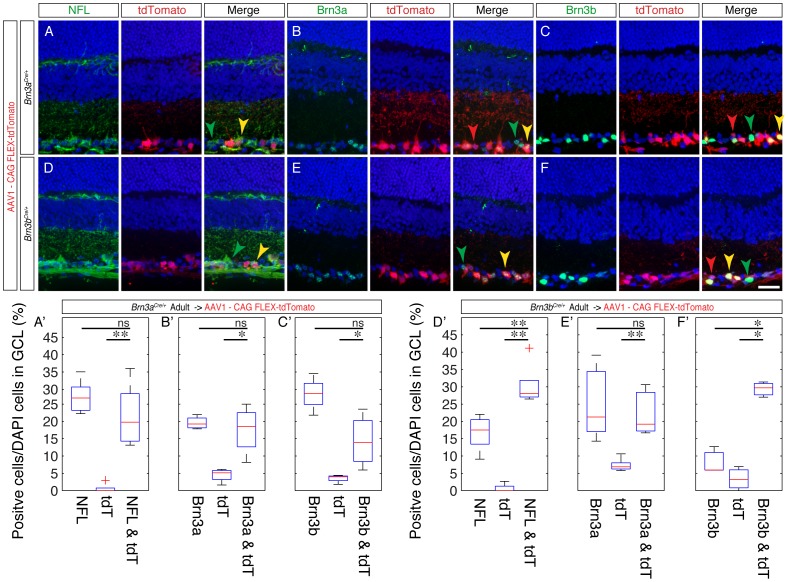
tdTomato positive cells in AAV1-FLEX-tdTomato infected *Brn3a^Cre/+^* and *Brn3b^Cre/+^* retinas express the RGC markers Neurofilament Light Chain (NFL), Brn3a and Brn3b. Eyes from *Brn3a^Cre/+^* or *Brn3b^Cre/+^* were infected with the Cre dependent AAV1-FLEX-tdTomato virus at P0, as shown in Figure 5. At p21, eye cups were prepared and briefly inspected under a fluorescence microscope for degree of infection. Highly infected eyes were then processed for sectioning and immunostaining. Sections from either *Brn3a^Cre/+^* (A–C) or *Brn3b^Cre/+^* (D–F) retinas were stained with antibodies against NFL (A, D), Brn3a (B, E) and Brn3b (C, F), shown in the left panels, in conjunction with endogenous tdTomato red fluorescence, shown in the middle pannels. Images were taken and quantitations performed on areas showing highest levels of infection, as judged by tdTomato fluorescence. Red, green and yellow arrowheads point at examples of red and green single positive or double positive cells. A’–F’ Box-whisker plots representing quantitations of experiments shown in A–F. For each experiment, the numbers of marker, tdTomato, or double positive cells were normalized to the total number of DAPI positive cells in the GCL. Significance levels shown were calculated with the Kolmogorov – Smirnov test for comparisons of two unknown distributions: ns = not significant, *p<0.05, **p<0.01 For number of quantitated images, cells counted, averages, standard deviations and significance levels by Kolmogorov-Smirnov and Student T tests, see Tables S1 and S2. Scale bar in F = 25 µm.

Given the surprising early ubiquitous expression of Cre from the newly generated *Brn3^CKOCre/+^* alleles and the *Brn3b^Cre/+^* derivative, we wanted to know whether Cre expression would reproduce Brn3 expression at later stages of development. Since transgenic reporters would likely be converted by the early recombination effect, we used a Cre dependent reporter delivered by an AAV vector (Fig. 5). The viral vector was AAV1.CAG.FLEX.tdTomato.WPRE.bGH (henceforth AAVFLEXtdT), using an AAV1 capsid known to infect many retinal neuronal cell types, including RGCs [49], containing a ubiquitously expressing chicken beta actin enhancer CMV promoter fusion (CAG), and a red fluorescent tdTomato cDNA [50] in reverse orientation, flanked by the arms of the FLEX cassette [51], and followed by a WPRE (a posttranscriptional regulatory element derived from a woodchuck hepatitis virus [52]) and bovine Growth Hormone polyadenilation signal. This virus has been previously used to report Cre expression in transgenic models [53], [54], and we tested its Cre dependent red fluorescence expression by infecting Cre-expressing HEK293 cells and appropriate controls (data not shown). We co-injected the AAVFLEXtdT together with a constitutively GFP expressing AAV1 virus (AAV1.CAG.GFP, henceforth AAVGFP) intra-vitreously in the eyes of either *Brn3b^Cre/+^, Brn3b^Cre/Cre^, Brn3a^Cre/+^,* or wild type (WT) P0.5 pups, and then tested their retinas for red and green fluorescence at postnatal day 3.5 (P3.5), 7.5 (P7.5) and in weaned adults (P21 and above) (Fig. 5A, B). Adult retinas of AAV1 injected *Brn3b^Cre/+^* and *Brn3a^Cre/+^* mice showed large numbers of tdTomato positive cells in the Ganglion Cell Layer (GCL) (Fig. 5 C, D, G, H). In *Brn3b^Cre/Cre^* retinas, in which the number of RGCs is greatly reduced as a result of loss of both copies of the Brn3b gene, tdTomato positive cells were still present, but in lower numbers compared to the *Brn3b^Cre/+^* mice, and we noticed a large number of looping axons at the periphery, consistent with previously reported RGC losses in these mice (Fig. 5 E, F). A few isolated tdTomato positive cells were observed in WT retinas (Fig. 5 I, J). The overall success of P0 eye injections is documented by the presence of GFP positive cells, which are infected by the Cre independent AAVGFP virus. Thus it can be easily appreciated from the green channel fluorescence of the retinal flat mounts that plenty of cells were infected in all retinas (Fig. 5C–J). Taken together, these results suggest that the *Brn3a^Cre^* and *Brn3b^Cre^* alleles can indeed specifically induce the Cre dependent AAVFLEXtdT reporter. Since essentially all tdTomato positive cells are localized to the GCL (Fig. 6C), and have characteristic axonal and dendrite arbor morphologies, (Fig. 6B, D and E), we propose that a large fraction are RGCs. Moreover, virtually all tdTomato positive cells observed in adult *Brn3a^Cre^* or *Brn3b^Cre^* retinas were also positive for the RGC marker NFL (Fig. 7A, A’ and D, D’, compare tdT only to tdT & NFL columns, Tables S1 and S2 for number of quantitated sections, counted cells and statistical significances), suggesting that the expression of the two Cre alleles is restricted to RGCs, at least in the early postnatal period. However, only about 50% (for Brn3a) and 66% (for Brn3b) of NFL positive cells were tdTomato positive. This could be due to incomplete infection, selective tropism of the AAV1 capsid, or restricted expression of the *Brn3^Cre^* alleles in subpopulations of RGCs [16], [55]. We then asked whether tdTomato cells labeled in either *Brn3a^Cre^* or *Brn3b^Cre^* retinas were indeed expressing the Brn3a and Brn3b proteins (Fig. 7 and Tables S1 and S2). Amongst tdTomato positive cells in *Brn3b^Cre^* retinas, about 90% were Brn3b positive (Fig. 7C, C’), whereas only about 80% of tdTomato positive cells in *Brn3a^Cre^* retinas were Brn3a positive (Fig. 7B, B’). Although a large majority of tdTomato positive cells generated by the Brn3a or Brn3b Cre drivers are indeed positive for the respective transcription factor in the adult, a substantive amount of cells are not. Since these cells became tdTomato positive presumably through Cre dependent recombination, the simplest explanation is that the expression domain of the *Brn3a^Cre^* and *Brn3b^Cre^* alleles, although restricted to RGCs, may be broader at early postnatal ages compared to the adult [56], [57]. Interestingly, the degree of overlap between tdTomato and Brn3a positive cells in *Brn3b^Cre^* retinas and tdTomato and Brn3b positive cells in *Brn3a^Cre^* retinas were also in the range of 70–80% of all tdTomato positive cells, in keeps with the known large overlap of Brn3a and Brn3b expression in RGCs during development and in the adult [16], [56], [57].

## Discussion

### Sequential Dre to Cre Recombination

To our knowledge, this is the first report using a combinatorial genetic strategy in which a Dre and a Cre recombinase are both required to activate a reporter gene. Previous analogous strategies have been used by crossing a Flp and Cre dependent reporter to Flp and Cre expressing mouse drivers with distinct patterns of expression [58], [59]. Our results show fully penetrant Dre recombination with essentially no cross-reactivity between Dre, Cre and their target sites. These results are very important, as they demonstrate the utility of Dre in combinatorial approaches. Placing the Dre recombinase at one genetic locus and intersecting it with a Dre dependent Cre recombinase expressed from another locus which will then target a reporter expressed from a third locus could theoretically allow us to target a specific cell population by the intersection of three specific gene expression patterns. Here we explore only the Dre and Cre recombination resulting from generally expressed Dre drivers and Cre reporters, but we hope in the future, we, and others will be able to develop more tissue or cell type specific alleles expressing either Dre or Dre dependent Cre expressing lines.

### Dre Independent Cre Expression from the Conditional *Brn3a^CKOCre^* and *Brn3b^CKOCre^* Alleles

The results in figures 3 and 4 show relatively high level of AP induction from the *Brn3^CKOCre/+^; ROSA26^iAP/+^*, suggesting a leaky expression of the Cre reporter in the absence of the Dre inducible endogenous Brn3 ablation. The targeted alleles for both *Brn3a^CKOCre^* and *Brn3b^CKOCre^* contain triple SV40 polyA transcription stop signals designed to prevent read through transcription from the endogenous Brn3 gene into the following Cre open reading frame. This strategy was used successfully in the previously published *Brn3a^CKOAP^*, *Brn3b^CKOAP^* and *Brn3c^CKOAP^* alleles [8], [16], [45], and the loci are identical except for the substitution of the loxP sites and the AP cDNA with roxP sites and a Cre cDNA. In the case of the *Brn3^CKOAP^* alleles, a moderate amount of read through can be detected even if no Cre is present, after prolonged AP staining. It may be that analogous amounts of Cre recombinase generated in the *Brn3^CKOCre^* alleles are enough to recombine the reporter, thereby amplifying the effect of read through transcription. Whatever the cause, this Dre independent Cre expression makes these alleles less useful for analysis by sparse recombination, so it will have to be addressed in future designs, perhaps by creating FLEX type cassettes for roxP sites. It is worth noting that the pattern of expression revealed by the read through is consistent with the endogenous expression profiles of the Brn3a and Brn3b genes and very rarely reveals other types of neurons or cellular populations.

### Early Developmental Expression of Brn3a and Brn3b

Previously only one report had documented expression of Brn3a and Brn3b in the germline [47], and both Brn3a and Brn3b KO mice develop normally throughout the intrauterine stages and Brn3b KO mice are viable and fertile, while Brn3a KO mice die at birth. However, microarray data sets from developing male and female gonad tissues, collected in the Genito Urinary Molecular Anatomy project (GUDMAP, http://www.gudmap.org/) or reported by Jameson et al, show expression of both Brn3a (Pou4f1) and Brn3b (Pou4f2) at several stages of male and female gonad development [60]–[62], so it is conceivable that enough Cre protein is deposited in the egg and the sperm to operate successful recombination upon fertilization. This expression profile might explain the whole animal recombination pattern we discovered in our triple transgenic animals, and which we confirmed at least for Brn3b using the derived *Brn3b^Cre^* knock-in line. It is worth noting that embryos in which the *Brn3a^CKOAP^* and *Brn3b^CKOAP^* alleles were induced using the ubiquitous *ROSA26^CreER/+^* driver typically show AP expression only in the later, expected expression domain of the Brn3 genes. The early embryonic or germ line expression of the Brn3s might hence be transient, however immortalized by virtue of the Cre dependent conversion of the ubiquitous reporter. This unexpected expression profile justifies the combinatorial approach we took, and hopefully be circumvented in the future by the generation of tissue specific or sparse recombination Dre drivers.

### Brn3 Loci as Drivers for RGC Specific Cre Lines

We demonstrate here that the *Brn3a^Cre^* and *Brn3b^Cre^* allele can successfully activate in a RGC dependent manner a fluorescent reporter delivered by an AAV virus. Since the Cre dependent AAV1-FLEX-tTomato virus is expressed only in *Brn3a^Cre^* and *Brn3b^Cre^* retinas but not WT controls, the labeled cell bodies are overwhelmingly restricted to the Ganglion Cell Layer, and sparsely or densely labeled cells have dendritic arbors with characteristic RGC morphologies, and axons tracking to the optic disc, and exhibit positivity for the RGC markers NFL, Brn3a or Brn3b, we are fairly confident that our alleles are exclusively expressed in RGCs in the early postnatal period. The fact that these are knock-in alleles, means that the mice in which they will be used will be heterozygotes for the Brn3a or Brn3b loci. However, both *Brn3a^Cre/WT^* and *Brn3b^Cre/WT^* lines are viable and fertile, and it is known for a long time in the field that RGCs which are heterozygote for either Brn3a or Brn3b are normal in numbers, gene expression and morphology and the mice exhibit normal visual reflexes [8], [16], [63]–[65], (and Kretschmer and Badea, unpublished observations), perhaps because loss of Brn3 alleles tends to increase the expresson of the remaining alleles through the removal of an autoregulatory loop [66] However it is still conceivable that subtle differences, which have not yet been reported, persist between Brn3 heterozygote and wild type cells.

This opens the possibility for delivering a variety of virally encoded reporters or reagents to the about 14 RGC types which express either Brn3a or Brn3b, essentially throughout all stages of RGC development and adults. We chose to infect P0 pups and follow the dynamics of expression over several time points in early postnatal development, and found fairly high levels of expression of the fluorescent reporter as early as three days after infection, allowing us to image dendritic arbors of individual RGCs with fairly high accuracy. We feel that the window of Cre expression from the Brn3 alleles is uniquely useful for targeting broad populations of Retinal Ganglion Cells. Several other transgenic, BAC transgenic or knock-in lines that target populations of RGCs have been generated. Prior to Brn3 expression, RGC precursors are labeled by the transcription factor Atoh7 (Math5), and a knock-in line as well as a BAC transgenic have been generated, both of which label a multitude of other cell types besides RGCs [67], [68], implying that the transcription factor might be needed but not sufficient for the RGC lineage. Additionally several knock-in, transgenic and BAC transgenic lines using elements of the JamB, Melanopsin, Parvalbumin, and P2 genes [49], [69]–[72] have been successfully used to label more or less restricted RGC subpopulations, typically at later onsets of development. Nevertheless, we believe that given the broad and early expression pattern, the *Brn3a^Cre^* and *Brn3b^Cre^* will be useful tools for delivering a variety of reporters and other tools to RGCs, beginning with early stages of development, as well as to other neuronal populations where Brn3s are expressed [45].

### AAV1-FLEX Vectors in Retinal Ganglion Cells

In this study we have used intravitreal injection of a Cre dependent red fluorescent AAV1 (AAV1-FLEX-tdTomato-WPRE-bGH) in conjunction with a constitutive, Cre independent green fluorescent AAV1 (AAV1-CMV-eGFP-bGH) in mouse retinas at postnatal day 0. Both viruses have the AAV1 capsid, and ubiquitous promoters, but the Cre dependent AAV1-FLEX-tdTomato-WPRE-bGH was not expressed in wild type retinas, and expressed selectively in RGCs in the *Brn3a^Cre^* and *Brn3b^Cre^* retinas, whereas the Cre independent AAV1-CMV-eGFP-bGH was expressed in multiple layers including the Ganglion Cell Layer, both in wild type and *Brn3a^Cre^* and *Brn3b^Cre^* retinas. These observations encourage us to conclude that, when delivered in early postnatal (P0) retinas, the AAV1 capsid is successfully (and quite quantitatively) targeting RGCs. AAV vectors can be classified based on sequence of the capsid encoding *cap* gene into at least 11 serotypes, and it appears that each serotype is characterized by distinct efficiencies of expression and retinal cell population target selection [73]–[75]. Early characterizations of virus tropism amongst different cell populations suggested that the AAV2 capsid might be preferentially targeted to RGCs, however several newer studies suggest that, depending on subretinal or intravitreal delivery, and on the retinal developmental stage at which the virus is delivered, many other serotypes perform well in RGCs [49], [76], [77], and that AAV1 capsid vectors can effectively infect RGCs [49], [77]. In these studies it is also shown that AAV1 positive cells tend to be seen much quicker (4–5 days after injection) compared to other serotypes [73]. We now report tdTomato positive cells in postnatal day 3.5 (P3.5) retinas infected at P0.5, however it is fair to assume that higher levels of expression of the reporter will accumulate over time. It is also not clear whether all cells of a retina are infected synchronously at the time of injection, and how many copies per cell are expressed, and hence how much protein will accumulate in each cell, determining when fluorescence would have reached detectable levels, however in most experimental scenarios we envision, the readout of the experiment would be performed in the adult stage, when hopefully expression would have saturated across samples.

The potential differential tropism of AAV capsids for different RGC types and/at different developmental stages, combined with the cell type distribution of Cre alleles expressed in broader or narrower RGC subpopulations, could be theoretically used to restrict AAV based RGC labeling and manipulation to specific cell types. Since infection efficiency will depend in a major fashion on the injection success, the absolute number of RGCs per retina infected by this strategy is less relevant, although the levels we observed are comparable to previously reported data, as referenced above. We find that, in sections from well infected areas of the retinal preparation about half of the RGCs are infected, as judged by double immunostaining with RGC markers. Since both Brn3b and Brn3a are expected to be expressed in about 75–85% of RGCs in the adult, this means that our strategy, under current optimal conditions, is missing about 25% of the adult target population. Thus, most but not all *Brn3a^Cre^* or *Brn3b^Cre^* RGCs can be targeted by P0 intravitreal injections of AAV1. However, most of our experimental paradigms will require much lower levels of infection, which will allow the identification and characterization of individual RGC types by dendritic arbor morphology. The incomplete targeting could depend on variable injection technique, temporally dynamic expression pattern of *Brn3^Cre^* alleles in Brn3 RGCs during development, or changes in virus tropism for different cell types at different ages. These complex interactions between Cre alleles, and virus tropism should be seen as opportunities for generating combinatorial strategies for targeting specific cell types by choosing a certain Cre expressing allele, an AAV of a specific serotype and promoter and a specific time window of delivery. These strategies could allow us to map the developmental expression domain of Brn3a and Brn3b onto the adult distribution of RGC types, with relevance to the cell autonomous or non-autonomous roles of Brn3s in RGC development.

## Supporting Information

Table S1 Quantitations for immunostaining experiments reported in Figure 7. Numbers of sections imaged, and cells counted for each image, together with average and standard deviation for each combination of genotype and marker (NFL, Brn3a and Brn3b) are provided.
(TIF)

Table S2 Statistical significance tests for comparisons presented in Figure 7. The Kolmogorov –Smirnov test for comparing two data samples of unknown distribution, and the Student T –test for comparing two distribution assumed to be normal were performed. For both tests, the null hypothesis states that the two data sets are drawn from the same distribution, or in other words, there are no differences between the compared conditions. A 0 indicates that the null hypothesis was not rejected, whereas a 1 indicates that the null hypothesis was rejected and the two samples are significantly different, with the corresponding p values.
(TIF)
