# Supplementary figures and images for: Dre - Cre Sequential Recombination Provides New Tools for Retinal Ganglion Cell Labeling and Manipulation in Mice
Source: PLoS One. 2014 Mar 7;9(3):e91435. doi: 10.1371/journal.pone.0091435 (PMC3946778; doi:10.1371/journal.pone.0091435)

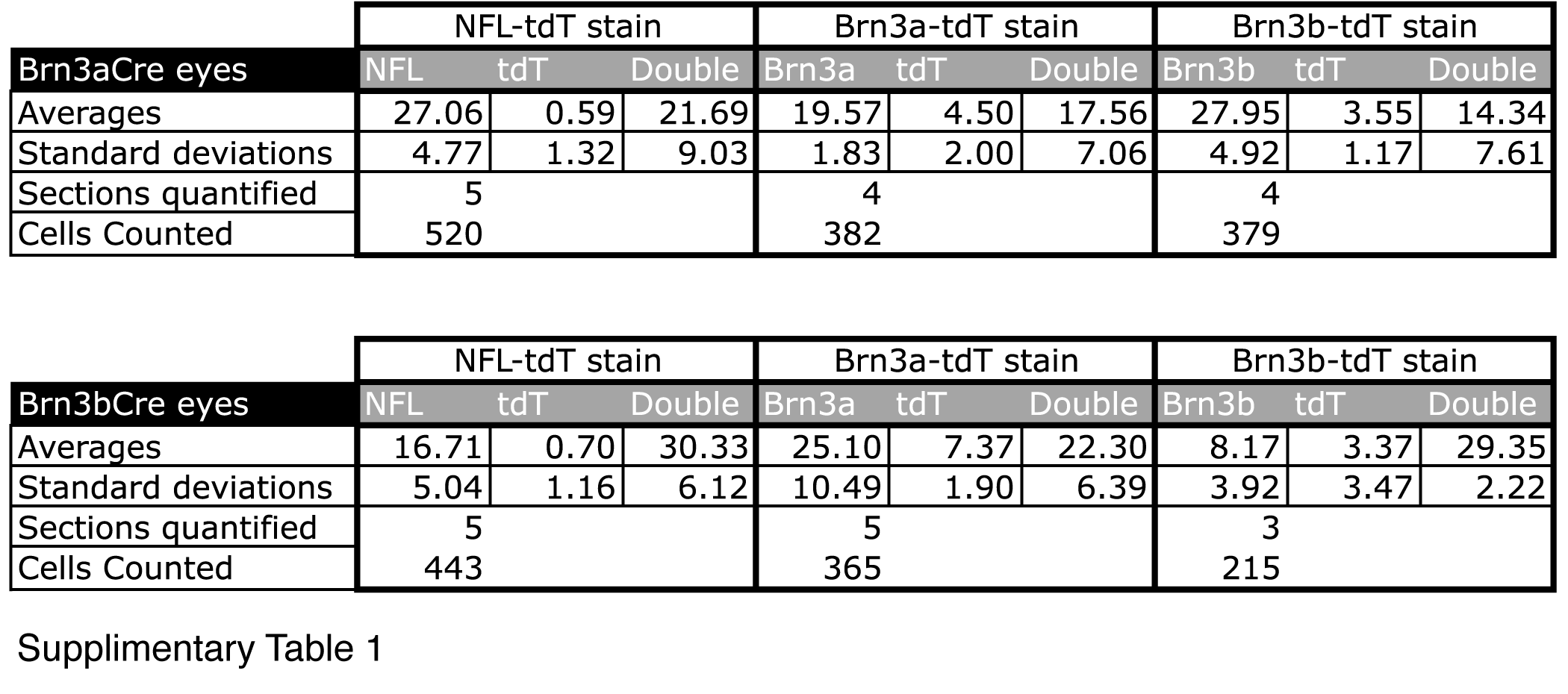

Supplement: Table S1 — Quantitations for immunostaining experiments reported in Figure 7. Numbers of sections imaged, and cells counted for each image, together with average and standard deviation for each combination of genotype and marker (NFL, Brn3a and Brn3b) are provided. (TIF) [file pone.0091435.s001.tif]

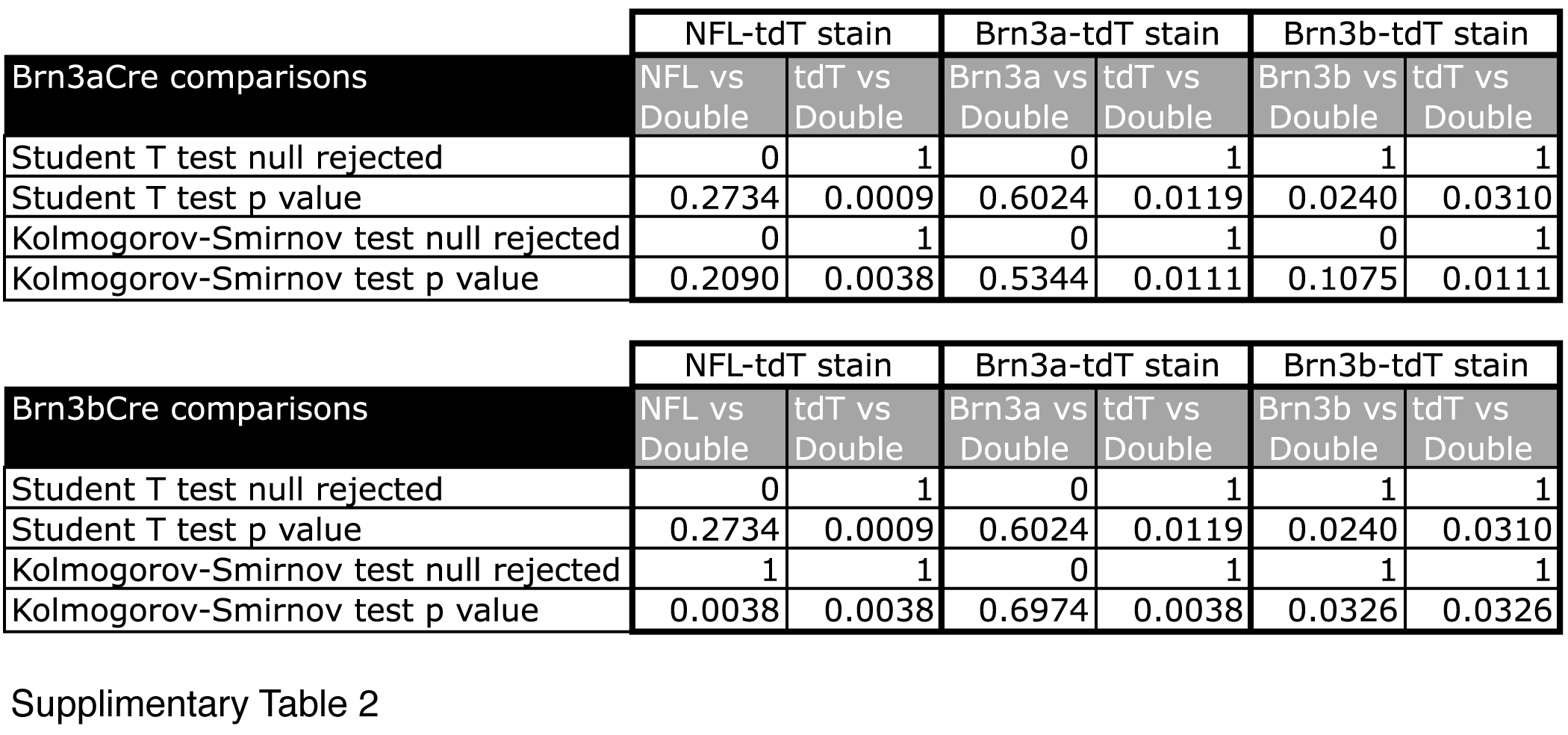

Supplement: Table S2 — Statistical significance tests for comparisons presented in Figure 7. The Kolmogorov –Smirnov test for comparing two data samples of unknown distribution, and the Student T –test for comparing two distribution assumed to be normal were performed. For both tests, the null hypothesis states that the two data sets are drawn from the same distribution, or in other words, there are no differences between the compared conditions. A 0 indicates that the null hypothesis was not rejected, whereas a 1 indicates that the null hypothesis was rejected and the two samples are significantly different, with the corresponding p values. (TIF) [file pone.0091435.s002.tif]
